# Integrative Mechanistic Studies Identify Reticulon-3 as a Critical Modulator of Infectious Exosome-Driven Dengue Pathogenesis

**DOI:** 10.3390/v17091238

**Published:** 2025-09-13

**Authors:** Razieh Bitazar, Clinton Njinju Asaba, Saina Shegefti, Tatiana Noumi, Julien Van Grevenynghe, Salim T. Islam, Patrick Labonté, Terence Ndonyi Bukong

**Affiliations:** 1 Armand-Frappier Santé Biotechnologie Research Center, Institut National de la Recherche Scientifique, Laval, QC H7V 1B7, Canada; razieh.bitazar@inrs.ca (R.B.); patrick.labonte@inrs.ca (P.L.); 2 PROTEO, The Quebec Network for Research on Protein Function, Structure, and Engineering, Montréal, QC H2X 3Y7, Canada

**Keywords:** reticulon 3, dengue, flavivirus, infectious exosomes, endoplasmic, multivesicular, trafficking, monocytes, immunoevasion, pathogenesis, antivirals

## Abstract

The dengue virus (DENV) exploits host cell exosome pathways to disseminate and evade immunity. However, the host factors enabling this process remain poorly defined. Here, we demonstrate that DENV infection robustly induces expression of the short isoform of Reticulon 3 (RTN3S) in hepatic (Huh7) and monocytic cells, and that RTN3S is a critical driver of infectious exosome biogenesis. RTN3S physically associates with double-stranded viral RNA and the DENV non-structural protein 3 (NS3) in infected cells, indicating its integration into the viral replication complex. Loss of RTN3 markedly reduced exosome production and the exosomal export of viral RNA and proteins, demonstrating that RTN3S is required for efficient exosome-mediated viral release. Conversely, overexpression of full-length RTN3S dramatically increased the release of infectious virus-containing exosomes; truncation of the RTN3S C-terminal domain abolished this enhancement, confirming the essential role of the C-terminus in RTN3S’s pro-viral exosomal function. In DENV-infected monocytes, we observed a shift toward a CD16-positive intermediate phenotype, accompanied by the upregulation of genes involved in vesicle biogenesis and stress response. These infected monocytes also secreted higher levels of inflammatory cytokines. Similarly, monocytes from Dengue patients exhibited high RTN3 expression, which correlated with an expansion of intermediate (CD16^+^) subsets and enriched expression of vesicle trafficking machinery genes. These findings reveal a previously unrecognized mechanism by which DENV hijacks RTN3S to promote the formation of infectious exosomes, thereby facilitating viral dissemination and immune evasion. RTN3S thus represents a novel element of the Dengue pathogenesis and a potential target for host-directed antiviral strategies.

## 1. Introduction

Dengue virus (DENV) infection has emerged as an urgent global health threat, with its incidence escalating at an alarming rate and its geographic footprint expanding steadily. Once confined largely to tropical regions, this infection now threatens populations across more than 125 countries [1,2]. The World Health Organization (WHO) reports an eightfold increase in annual DENV cases over the past two decades, estimating 100–400 million infections each year and placing about half of the world’s population at risk [3]. Multiple converging factors are suggested to drive this surge. These include rapid urbanization and global population growth that have created dense human–mosquito contact zones. At the same time, increased international travel and trade have helped ferry *Aedes* mosquito vectors and DENV across borders [4]. Moreover, warmer climate changes characterized by altered rainfall and humidity are extending the habitat range of *Aedes aegypti* and *Aedes albopictus* mosquitoes, opening the door for DENV transmission in previously unaffected areas, including parts of the United States, Canada, and Europe [4,5]. The result is a burgeoning global DENV infection burden that can potentially strain public health systems and require new strategies to curb its spread. Yet, no specific antiviral therapy or broadly effective vaccine exists to counter DENV [6], making it imperative to deepen our understanding of Dengue pathogenesis as a foundation for novel interventions.

Dengue’s clinical spectrum ranges from mild to life-threatening hemorrhagic fever [7], largely shaped by the complex interplay between the virus and the host immune system. A hallmark of DENV pathogenesis is its proclivity to infect and replicate within immune cells that would ordinarily coordinate antiviral defenses. Monocytes, macrophages, dendritic cells (DCs), and even B and T lymphocytes are all targets for DENV [6,8]. By seeding itself in these cells, DENV actively subverts immune responses: the infection of DCs and monocyte-lineage cells impairs their antigen presentation and cytokine production, dysregulating antiviral functions and facilitating viral dissemination [9,10]. In essence, the virus turns the body’s defenders into Trojan horses. Concurrently, DENV has evolved mechanisms to evade innate immune detection and antiviral signaling. Its nonstructural proteins (like NS2A, NS4A, NS4B, and NS5) blunt the type-I interferon response, a key early antiviral defense [11,12], by targeting critical signaling molecules. For example, DENV can prevent phosphorylation of STAT1 (via NS2A/NS4A/NS4B) and even induce degradation of STAT2 (via NS5) [13,14,15], thereby antagonizing interferon pathways and allowing the virus to replicate unabated in host cells. This immune evasion is further compounded in secondary infections by antibody-dependent enhancement (ADE) [16], wherein non-neutralizing antibodies from a prior DENV exposure facilitate increased infection of Fc-receptor-bearing cells, often exacerbating disease severity. Together, the ability of DENV to cripple innate antiviral signaling and exploit the host’s immune cells underlies the uncontrolled viral replication and hyperinflammatory cascades seen in severe Dengue cases.

Amid these virus–host dynamics, an intriguing role has emerged for extracellular vesicles, particularly exosomes, in DENV pathogenesis [17,18,19,20]. Exosomes are nanoscale vesicles released by cells, capable of ferrying proteins, RNA, and other biomolecules between cells. During DENV infection, mosquito vectors and human host cells secrete exosomes laden with viral material. Remarkably, exosomes from DENV-infected cells have been found to contain the *full-length viral genome* and viral proteins [21,22], rendering them infectious to new target cells. These virus-packed exosomes can shuttle DENV between cells covertly, effectively forming a hidden transmission route that shields the virus from neutralizing antibodies and immune surveillance [23]. By altering the cargo and even the size of exosomes (DENV-induced exosomes tend to be larger, presumably to accommodate the entire genome), the virus ensures its successful transfer and persistence in the host. The immunomodulatory effects of these vesicles are also under intense scrutiny: exosomal cargo from DENV-infected cells (such as specific microRNAs, cytokines, and even soluble NS1 protein) can modulate recipient cells’ immune responses [24], skewing them in ways that may promote virus survival or contribute to vascular leak and other pathogenic outcomes [24,25].

Considering DENV’s reliance on host cell machinery for both replication and stealth, host factors that mediate these processes have attracted growing interest. One such factor is Reticulon 3 (RTN3)**,** an endoplasmic reticulum (ER)-associated protein that has recently been implicated in the life cycles of several flaviviruses [26]. RTN3 belongs to a family of ER membrane-shaping proteins and is widely expressed in human tissues. Intriguingly, RTN3 appears to be hijacked by flaviviruses to facilitate the formation of viral replication organelles [26]. A recent study demonstrated that RTN3 (specifically the RTN3.1A isoform) is required for efficient replication of West Nile virus, Zika virus, and DENV, likely through a direct or indirect interaction with the viral NS4A protein [26]. NS4A is a DENV nonstructural protein known to remodel ER membranes and create vesicle packets where viral RNA replication occurs [26,27]. RTN3, as an ER membrane protein, may serve as a cofactor for NS4A’s membrane-bending activities [27]. Consistently, the absence of RTN3 was shown to trigger the degradation of viral NS4A and disrupt the assembly of DENV replication complexes, resulting in impaired production of new viral particles. Beyond supporting replication, RTN3 has also been linked to the *exosome-mediated* phase of viral infection. Our recent studies with the hepatitis C virus (HCV)—another positive-strand RNA virus in the Flavivirus family—revealed that RTN3 is upregulated during infection and is incorporated into exosomes carrying infectious viral RNA [28]. Knocking down RTN3 in HCV-infected cells significantly reduced the release of infectious virus-containing exosomes, whereas RTN3 overexpression enhanced it. These findings led us to conclude that RTN3 acts as a key regulator of viral exosome loading and release, effectively helping to smuggle viral genomes out of the cell under the radar of the immune system [28]. By analogy, in the context of Dengue, RTN3 might play a dual role: (i) assisting DENV replication by stabilizing critical viral proteins and remodeling membranes, and (ii) facilitating the packaging of DENV RNA into exosomal vesicles for cell-to-cell transmission. This emerging picture places RTN3 at the intersection of two central facets of the DENV life cycle—intracellular replication and intercellular spread—making it a particularly compelling subject for further research.

In all, existing evidence indicates that RTN3 plays a critical role in DENV pathogenesis; however, its precise function in the generation and/or loading of infectious exosomes remains unresearched. To date, no study has definitively shown whether or how RTN3 contributes to the assembly of these infectious DENV exosomes, revealing a pressing gap in our understanding. Our current research seeks to clarify if and how RTN3 might influence or modulate infectious exosome generation, potentially unveiling new antiviral strategies.

## 2. Materials and Methods

### 2.1. Huh7 Cell Culture, Infection, and Co-Culture Experiments

Huh7 hepatoma cells were cultured in Dulbecco’s Modified Eagle Medium (DMEM) (ThermoFisher Scientific, Cleveland, OH, USA) supplemented with 1% penicillin/streptomycin (P/S) (10,000 U/mL penicillin, 10,000 µg/mL streptomycin; Gibco, REF.NO. 15140-122), 10 mM HEPES buffer (Gibco, REF.NO. 15630-080), and 10% exosome-depleted FBS (Gibco; Cat.NO. A2720801). For infection, 2.5 × 10^5^ cells were seeded in 6-well plates and infected with DENV-2 (New Guinea C strain (ATCC VR-1584^TM^) at a multiplicity of infection (MOI) of 0.1. After 1 h of incubation at 37 °C with 5% CO_2_, unbound virions were removed, and cells were maintained for 72 h. Supernatants were collected and centrifuged at 500× *g* for 10 min to remove cell debris. These were used for downstream plaque assay and exosome isolation. Cells were harvested for RNA and protein extraction. For co-culture experiments, 2.5 × 10^5^ Huh7 cells were seeded and treated with titrated infectious exosomes at MOI 0.1, incubated for 72 h, and used for downstream RNA, protein extraction, Western blotting, and qPCR analyses.

### 2.2. THP-1 Cell Culture, Infection, and Flow Cytometry Analysis

The THP-1 human monocytic leukemia cell line was cultured in RPMI1640 medium (ThermoFisher Scientific, Cleveland, OH, USA) supplemented with 10% exosome-depleted fetal bovine serum (Gibco; Cat.NO. A2720801), 1% penicillin/streptomycin (P/S) (10,000 U/mL penicillin, 10,000 µg/mL streptomycin; Gibco, REF.NO. 15140-122), 10 mM HEPES buffer (Gibco, REF.NO. 15630-080). For infection experiments, 1 × 10^6^ cells were seeded in 6-well plates and infected with DENV-2 at an MOI of 5. Cells were incubated for 24-, 48-, and 72 h post-infection. Supernatants were harvested and clarified by centrifugation at 500× *g* for 10 min to remove debris, followed by storage at −80 °C for exosome extraction and NanoFCM analysis, or at −150 °C for viral titration. Cells were harvested for downstream RNA and protein extraction. Flow cytometry was performed to assess viability and infection efficiency. Cells were stained with Fixable Viability Stain 7-AAD (BD Biosciences Cat# 555815) and antibodies against CD14 (BD Horizon Cat # 563419), CD16 (Biolegend Cat # 302012), and intracellular NS3 (GeneTex Cat # GTX124252). Flow cytometry data were analyzed using FlowJo software v10.7.1.

### 2.3. Exosome Isolation and Characterization

Conditioned media from THP-1 monocytes and Huh7 hepatocytes, uninfected or DENV-infected, were collected at the indicated times, cleared at 500× *g* for 10 min and 2000× *g* for 10 min, and subjected to immunomagnetic positive selection (EasySep™ Human Pan-Extracellular Vesicle kit, Stemcell #17891, Vancouver, BC, Canada) to capture CD9/CD63/CD81-positive exosomes. For all downstream assays, we used the immunoselected fraction only, which achieved 100% CD63^+^ purity by NanoFCM after immunoselection. To exclude non-vesicular and free-viral RNA, purified exosomes were treated with RNase before use in co-culture infection assays, immunophenotyping, and protein profiling. For nano-flow cytometry, EV aliquots from all four conditions (THP-1 and Huh7; uninfected and DENV-infected) were analyzed on a Flow NanoAnalyzer (NanoFCM N30E, Xiamen, China) calibrated with 250 nm silica beads and a 68/91/113/155 nm sizing beads. Samples were diluted in 1× DPBS (DPBS negative control) and acquired under standardized settings (laser 10 mW, side-scatter decay 10%, sampling pressure 1 kPa) at 2000–12,000 events/min. Size and concentration were computed in NanoFCM Profession v2.31 using a 40–200 nm gate (>99% of detected particles) and reported as particles per mL of the original sample. All measurements were performed in triplicate. For fluorescence phenotyping, EVs (~2 × 10^11^ particles/mL) were stained with AF647 anti-human CD63 (NanoFCM, China, Cat.No.NHA009-A647-15T) in 10 mM HEPES, diluted in low-salt TE to limit nonspecific interactions, mixed by adding 10 µL of 1:50 prediluted antibody to 50 µL EVs, diluted 1:500 in acquisition buffer, and incubated 1.5 h at 37 °C in low-bind microtubes (Axygen^®^, Corning, NY, USA, Cat.No.MCT-060-L-C), protected from light with gentle shaking. Unstained CCS, antibody-only, and uninfected biological controls were used to set gates and confirm specificity, and the same instrument settings were used for all acquisitions.

### 2.4. Plaque Assay

Viral titers of infectious exosomes were determined by plaque assay on Vero cells seeded at 2.5 × 10^5^ cells/well in 24-well plates. Monolayers were infected with 10-fold serial dilutions of exosome preparations in 0.2 mL volumes, adsorbed for 1 h at 37 °C with gentle rocking every 15 min. A 1 mL overlay of MEM-CMC was applied, and cells were incubated at 37 °C with 5% CO_2_ for 5 days. Plates were fixed with 10% formaldehyde, stained with crystal violet, and plaques counted to calculate PFU/mL.

### 2.5. Protein Extraction and Immunoblotting from Cells and Exosomes

THP-1 and Huh7 cells, either uninfected or infected with DENV, were washed with ice-cold PBS and lysed on ice using RIPA buffer containing protease inhibitors (cOmplete™, Mini; Roche Applied science, Indianapolis, IN, USA, Cat. # 11836153001). The RIPA buffer had a standard composition (~50 mM Tris-HCl at pH 7.4; 150 mM NaCl; 1% NP-40 or Triton X-100; 0.5–1% sodium deoxycholate; and 0.1% SDS). Lysates were incubated on ice for 30 min with occasional gentle mixing, briefly vortexed, then kept on ice for another 5 min before being centrifuged at 14,000 rpm (Eppendorf 5417R centrifuge; ~17,750× *g*) for 20 min at 4 °C. The resulting supernatants were collected, total protein levels measured using a BCA assay, and aliquots stored for later immunoblotting.

Exosomes (200–300 µL per preparation), purified as described above and previously [28,29], were lysed on ice using the same RIPA buffer with inhibitors. The process involved 30 min of incubation with gentle mixing, a short vortex, an additional 5 min ice incubation, and centrifugation at 14,000 rpm (Eppendorf 5417R centrifuge, ~17,750× *g*) for 20 min at 4 °C. The supernatants containing exosomal proteins were then analyzed via BCA and subjected to Western blotting. Equal amounts of protein were separated using SDS-PAGE (either 8% or 12% gels, depending on the target) and transferred to PVDF membranes. RTN3 long and short isoforms were identified using ProteinTech (Cat. # 12055-2-AP) and Bethyl (Cat. # A302-860A) antibodies; DENV NS3 was detected with GeneTex antibody (Cat. # GTX124252). Exosomal markers CD63 (Santa Cruz Cat. # sc-365604) and HSP70 (Abcam Cat # Ab181606) confirmed the identity of exosomes, while calnexin (Abcam Cat. # Ab181606) served as a negative marker to rule out ER contamination.

### 2.6. CRISPR/Cas9 Knockdown and Lentiviral Transduction

RTN3S-specific gRNAs were cloned into the LentiCRISPRv2 vector (Addgene plasmid #52961, Feng Zhang; http://n2t.net/addgene:52961; RRID:Addgene_52961 (accessed on 11 September 2025)). HEK293T cells were co-transfected with psPAX2, pMD2.G, and LentiCRISPRv2-sgRNA plasmids. Viral supernatants were collected, filtered (0.45 μm), and stored at −80 °C. For titration, HeLa cells were infected with serial dilutions and selected with puromycin (1 µg/mL). Surviving colonies were stained with crystal violet and counted. Huh7 cells were transduced with lentivirus at MOI 5–10 in the presence of 8 μg/mL polybrene. Supernatants were centrifuged, and exosomes were quantified by NanoFCM.

### 2.7. Overexpression Plasmid Transfection

Huh7 cells were transfected using FUGENE 4K (Promega, Madison, WI, USA) with plasmids encoding RTN3S full-length or truncation mutants (ΔN11, ΔN45, ΔC36) in the Flag-CMV-2 backbone (gift from Prof. Mitsuo Tagaya) [30]. Transfection was followed by infection with DENV. Supernatants were collected and centrifuged to remove debris. Total particle and exosome counts were assessed by NanoFCM.

### 2.8. RNA Immunoprecipitation (RIP) and Co-Immunoprecipitation Analysis

Infected Huh7 cells samples were fixed at room temperature with 4% formaldehyde buffered saline for 10 min to initiate covalent crosslinking of RNA–protein complexes. Crosslinking was quenched by adding 1/10 volume of 1.25 M glycine (MULTICELL Cat. # 800-045) to reach a final concentration of 125 mM, followed by incubation at room temperature for 5 min. Cells were then washed twice with ice-cold PBS, scraped into cold PBS, transferred to tubes, and pelleted by centrifugation at 500× *g* for 5 min at 4 °C. All subsequent steps were performed on ice or at 4 °C to preserve RNA–protein interactions. The cell pellet was resuspended in 500 µL of lysis buffer (50 mM Tris-HCl pH 8.0 (0.060 g), 150 mM NaCl (0.088 g), 0.5% sodium deoxycholate (0.050 g), 0.1% SDS (0.010 g), 1% NP-40 (*v*/*v*; 100 µL), 1 mM EDTA (0.0037 g) with 1x protease and RNase inhibitor cocktail. Total cellular proteins were pre-cleared with protein G beads. 100 μg of total protein was incubated with anti-dsRNA (GenScript, Piscataway, NJ, USA, Cat. # A02181-40) and RTN3 (ProteinchTech, Rosemont, IL, USA, Cat. #12055-2-AP) antibodies. Immunoprecipitation was performed overnight at 4 °C using 1 in 100 dilutions of primary antibody and normal rabbit/mouse IgG (Santa Cruz cat #sc-3877 and sc-69786), non-specific antibody serving as IP control. A mixture of Protein A/G PLUS-Agarose beads (Santa Cruz cat. #sc-2003) was added, and the incubation was continued for an additional 60 min. The samples were washed with SDS ChIP lysis buffer supplemented with protease inhibitor and RNase inhibitor. The immunoprecipitants (protein-RNA complexes) were either used for Western blot analysis or RNA purification using the RNeasy Mini kit (Qiagen, Cat No. 74104, Hilden, Germany)

### 2.9. Quantitative Real-Time PCR Assay for DENV Genomic RNA and Host Gene Transcripts

Cell pellets and purified exosome fractions were lysed, and total RNA was recovered using the RNeasy Micro Kit (Qiagen, Cat No. 74104, Germany) according to the supplier’s instructions. One microgram of RNA from each sample was converted to cDNA with the iScript™ Reverse Transcription Supermix (Bio-Rad, Hercules, CA, USA). The resulting cDNA served as a template for SYBR Green-based quantitative PCR (qPCR) on a Bio-Rad CFX96 instrument. Primer pairs and cycling parameters were identical to those reported previously [28], and the full primer list is provided in Table 1. The assay simultaneously quantified DENV genomic RNA and the mRNA levels of selected host genes of interest. Relative expression was calculated with the delta-delta Ct method, normalizing each target to 18S rRNA as an internal control, as outlined in earlier studies [28].

### 2.10. Single-Cell Transcriptomic Analysis

We retrieved publicly available single-cell RNA sequencing (ScRNA-seq) data from peripheral blood mononuclear cells (PBMCs) of healthy individuals (C), Dengue patients (D), Dengue warning signs (DWS), and severe Dengue patients (SD). These datasets were obtained from the Gene Expression Omnibus (GEO) database, which is owned and operated by the National Center for Biotechnology Information (NCBI), a part of the National Library of Medicine (NLM) at the National Institutes of Health (NIH) in the United States. The GEO database is a public archive and resource for gene expression data. Specifically, we used datasets with accession code GSM6833297. A total of 4 healthy controls, 8 Dengue, 4 DWS, and 8 SD patients were included. Analyses were performed using Seurat (v5.1.0). Cells expressing 200–5000 genes, <40,000 counts, and <5% mitochondrial transcripts were retained (n = 154,220). Data was normalized (LogNormalize), top 2000 variable genes identified, and PCA was applied (top 20 PCs). UMAP and clustering (resolution = 0.4) followed. Cell types were annotated using SingleR and the Human Primary Cell Atlas. For monocyte re-clustering (n = 27,169), standard preprocessing was repeated. Differential gene expression between groups was determined using the Wilcoxon test (*p* < 0.05, avg_logFC threshold applied).

### 2.11. Data Availability

All data files and complete experimental details supporting this study are contained in the manuscript and its Supplementary Files. Any additional information will be provided promptly upon reasonable request to the corresponding author, provided such sharing does not compromise participant confidentiality, institutional or third-party agreements, ongoing reviews or filings, intellectual property considerations, or otherwise adversely affect the authors’ rights and obligations.

### 2.12. Ethics Statement

In this research, we performed a secondary analysis using a publicly available dataset. Therefore, the institutional review board (IRB) and ethical approval were not required, as the participant data had already been anonymized and made publicly accessible. Consequently, there were no additional ethical concerns regarding participant confidentiality or consent for this study.

## 3. Results

### 3.1. DENV Infection Upregulates RTN3 Isoforms and Exosome Secretion

**Figure 1:**
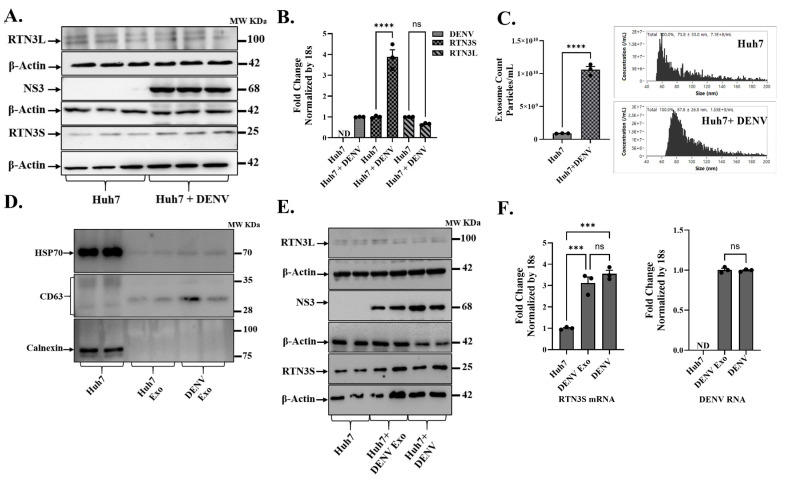
**Dengue virus infection upregulates RTN3 isoforms and promotes exosome release in Huh7 cells.** (**A**) Western blot analysis of Huh7 cell lysates without or with Dengue virus (DENV) infection. Membranes were probed with antibodies for RTN3 long (RTN3L, ~100 kDa) and short (RTN3S, ~25 kDa) isoforms, Dengue NS3 (~68 kDa), and β-Actin (~42 kDa) as a loading control. (**B**) RT-qPCR quantification of RTN3L and RTN3S mRNA in Huh7 cells ± DENV, normalized to 18S rRNA. The label ND denotes ‘not detected’. (**C**) NanoFlow Cytometry analysis (NanoFCM) of purified exosomes from Huh7 culture supernatants. **Left:** size-distribution histograms (30–150 nm) for exosomes from mock or DENV-infected cells. **Right:** bar graph of exosome concentration (particles/mL). (**D**) Western blots of whole-cell lysate (Huh7) and purified exosome fractions (Huh7 (whole cell lysate), Huh Exo, DENV Exo). Huh Exo are exosomes from uninfected Huh7 cells; DENV Exo are exosomes from DENV-infected Huh7 cells. Exosomal markers HSP70 and CD63 were assessed, whereas the ER protein Calnexin was probed to ascertain the purity of the exosome preparation. (**E**) Huh7 cells infected with DENV and Huh7 cells treated with purified DENV-derived exosomes (+ DENV Exo). Cell lysates were western blotted, then probed for RTN3L/S, DENV NS3, and β-Actin. (**F**) RT-qPCR quantification of DENV genomic RNA and RTN3S mRNA in Huh7 cells treated with DENV exosomes (+Exo) or with active DENV infection, normalized to 18S rRNA. Data in panels (**B**,**C**,**F**) represent mean ± SEM of ≥3 independent experiments; statistical significance was determined by Student’s *t*-test (* *p* < 0.05, ** *p* < 0.01, *** *p* < 0.001, **** *p* < 0.0001; ns = not significant).

Flaviviruses can remodel the endoplasmic reticulum (ER) into replication organelles and exploit endosomal trafficking to release infectious exosomes carrying viral genomes. This vesicular route can aid in subverting extracellular immunity and enables receptor-independent viral spread. Recently, we showed that the ER-shaping protein Reticulon-3 (RTN3), particularly its short isoform (RTN3S), can modulate flavivirus replication complexes and drive exosomal export of infectious hepatitis C virus RNA. Whether the Dengue virus similarly recruits RTN3S or other RTN3 variants to regulate infectious exosome biogenesis remains unknown. In human Huh7 cells, DENV infection induced a pronounced increase in the expression of the short isoform of RTN3 (RTN3S) protein (Figure 1A and Figure S1A), with a concurrent moderate change in the long isoform (RTN3L) (Figure 1A and Figure S1B). Western blots showed that RTN3S (~25 kDa) levels were low or barely detectable in uninfected cells but became upregulated upon infection, whereas RTN3L (~100 kDa) exhibited no change in expression. The viral NS3 protein (68 kDa) was readily detected only in infected cells, confirming active DENV replication (Figure 1A). Consistent with protein data, RT-qPCR (Figure 1B) indicated that DENV infection drove a significant rise in RTN3 mRNA levels. In infected Huh7 cultures, RTN3 transcripts (Figure 1B) precisely short isoform, were elevated ~3–4-fold relative to uninfected controls (*p* < 0.0001), while DENV genomic RNA was abundant only in infected cells (undetectable in controls).

Concomitant with RTN3 induction, DENV infection stimulated the biogenesis and secretion of exosomes. Nano flow cytometry (NanoFCM) revealed that DENV-infected Huh7 cells released significantly more extracellular vesicles (exosomes) than uninfected cells (1.5 × 10^9^ vs. 0.7 × 10^9^ particles per mL, *p* < 0.0001). The modal diameter of secreted vesicles also shifted from ~74 nm in controls to ~88 nm upon infection, suggesting slightly larger vesicles in the infected condition (Figure 1C). Nano-flow cytometry analysis of the supernatant from DENV-infected Huh7 cell cultures revealed a heterogeneous particle population, with only a small fraction being CD63^+^ (Figure S2A,B). After CD63-based immunoselection and RNase treatment, the sample displayed a uniform population consisting entirely of CD63^+^ particles (P1), with CD63^−^ events no longer present. Control samples that were either unstained or uninfected verified the accuracy of the gating, confirming the isolation of purified exosomes (Figure S2C). Exosome fractions from both uninfected and DENV-infected cells were enriched in HSP70 and CD63 (classical exosome markers), whereas calnexin (an endoplasmic reticulum protein) was undetectable in these fractions (Figure 1D). The absence of calnexin alongside the presence of HSP70/CD63 confirmed that the preparations consisted of bona fide exosomes without other cellular contamination. Notably, NS3 viral protein was found associated with exosomal fractions from infected cultures but not from uninfected controls (Figure 1E).

We next asked whether exosomes derived from DENV-infected cells (DENV-derived exosomes) could transfer viral material to naive recipient cells. Naïve Huh7 cells were incubated with purified exosomes from DENV-infected donor cells, and viral uptake was assessed in comparison to direct virus infection. Remarkably, cells receiving DENV-derived exosomes showed substantial levels of DENV RNA after 72 h, comparable to those in free DENV-infected cells (Figure 1F, right). DENV RNA remained undetectable in cells given exosomes from uninfected donors (negative control). The exosome-treated cells also contained DENV NS3 protein at 72 h post-treatment (Figure 1E). Intriguingly, co-culture of naïve Huh7 cells with DENV exosomes also recapitulated the effect of free virus on RTN3 expression in infected cells. Cells treated with DENV exosomes upregulated RTN3S mRNA to a similar extent (~3-fold increase) as cells infected with DENV itself (Figure 1F, left).

### 3.2. RTN3 Is Required for Virus-Induced Exosome Release and Viral Cargo Loading

**Figure 2:**
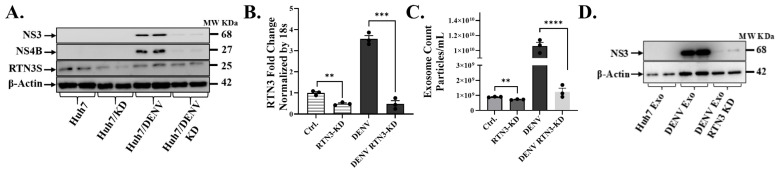
**RTN3 knockdown abrogates RTN3S expression and inhibits Dengue-induced exosome release in Huh7 cells.** (**A**) Western blot of Huh7 cell lysates after transfection with control no guide RNA CRISPR-Cas9 (Ctrl) or RTN3-targeting guided RNA CRISPR-Cas9 (RTN3-KD) followed by DENV infection. Blots were probed for DENV NS3 (~68 kDa), NS4B (~27 kDa), RTN3S (~25 kDa, arrow), and β-Actin (~42 kDa). (**B**) RT-qPCR of RTN3 mRNA in Huh7 cells (Ctrl or RTN3-KD, ±DENV), normalized to 18S rRNA. (**C**) NanoFCM analysis of exosome release for each condition. Control and RTN3-KD cells without infection release few exosomes (ns). (**D**) Western blot of exosome preparations from Ctrl (mock), DENV, and DENV + RTN3-KD conditions. Probing for DENV NS3 shows that exosomes from infected cells contain NS3, and this viral cargo is strongly diminished when RTN3 is knocked down. β-Actin is shown as a control. Data (**B**,**C**) are mean ± SEM of ≥3 experiments; significance by ANOVA or *t*-test (* *p* < 0.05, ** *p* < 0.01, *** *p* < 0.001, **** *p* < 0.0001; ns = not significant).

To determine the functional role of RTN3 in DENV infection and exosome production, we next employed RTN3 loss-of-function approaches. Huh7 cells were subjected to RTN3 knockdown (KD) using CRISPR-Cas9 before infection. The impact on viral replication and exosome release was assessed (Figure 2). Efficient RTN3 silencing was confirmed by a ~50% reduction in basal RTN3S transcript levels compared to control cells (Figure 2A). Notably, DENV infection normally caused a robust ~3–4-fold upregulation of RTN3 mRNA, this induction was completely abrogated in RTN3-KD cells (Figure 2B). RTN3-KD infected cells showed only minimal RTN3 transcript levels (down to ~10% of infected controls, *p* < 0.001), indicating sustained knockdown despite the infection-triggered response. Notably, RTN3 knockdown resulted in a significant impairment of DENV replication and exosomal virus export. Western blot analysis revealed that DENV NS3 and NS4B proteins were markedly reduced in RTN3-deficient cells compared to RTN3-competent infected cells (Figure 2A). In control infected cells, NS3 and NS4B accumulated to high levels, whereas in RTN3-KD infected cells, their expression was visibly diminished (NS3 band intensity reduced by ~40–50%). Importantly, the knockdown of RTN3 blunted the dramatic increase in exosome secretion normally driven by DENV. NanoFCM quantification showed that while infected control cells released an average of ~1 × 10^10^ particles/mL (a ~5-fold increase over uninfected baseline, *p* < 0.0001), infected RTN3-KD cells produced only ~1.5 × 10^9^ particles/mL; a level only modestly above uninfected controls and about 65% lower than wild-type infected cells (Figure 2C). In addition to quantity, RTN3 knockdown also affected the incorporation of viral components into exosomes. Exosomal fractions isolated from RTN3-deficient infected cells contained substantially less DENV NS3 protein than exosomes from RTN3-intact infected cells. By Western blot, NS3 was readily detected in exosomes derived from DENV-infected control cells, whereas exosomes from DENV-infected RTN3-KD cells showed only faint NS3 signals (Figure 2D).

### 3.3. RTN3S Overexpression and Truncated Mutants Differentially Affect Exosomal Viral Packaging and Infectivity

**Figure 3:**
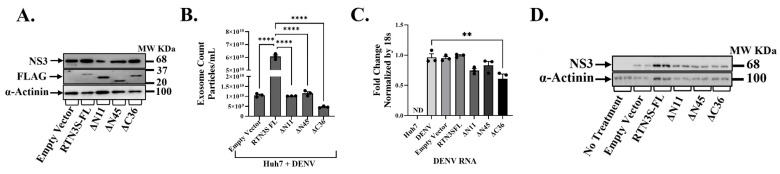
**The C-terminal domain of RTN3S is required for exosome production and viral transfer.** (**A**) Western blot of Huh7 cells infected with DENV and transfected with empty vector (EV), full-length FLAG-RTN3S (FL), or deletion mutants (ΔN11, ΔN45 remove N-terminal regions; ΔC36 removes C-terminal tail). Membranes were probed with anti-FLAG to detect RTN3S constructs (all ~20–37 kDa) and anti-DENV NS3 (~68 kDa); β-Actin (~42 kDa) is a loading control. (**B**) NanoFCM quantification of exosome release under each condition. The bar graph shows exosome concentration (particles/mL) for EV, RTN3S-FL, ΔN11, ΔN45, and ΔC36. Data are mean ± SEM (n = 3). (**C**,**D**) Huh7 cells were co-cultured with exosomes isolated from DENV-infected Huh7 donor cells transfected with either the empty control vector or the indicated RTN3 constructs. The experimental groups included untreated control cells (No Treatment) as well as cells co-cultured with exosomes derived from DENV-infected donor cells expressing RTN3 plasmid variants (Empty Vector, Exo-RTN3S-FL, Exo-ΔN11, Exo-ΔN45, and Exo-ΔC36). After co-culture, total RNA and protein were extracted from recipient Huh7 cells. Viral RNA levels were measured by RT-qPCR and normalized to 18S rRNA (**C**), while DENV NS3 protein (~68 kDa) was analyzed by Western blotting, with α-Actinin (~100 kDa) used as a loading control (**D**). Data (**B**,**C**) are mean ± SEM of ≥3 experiments; significance by ANOVA or *t*-test (* *p* < 0.05, ** *p* < 0.01, *** *p* < 0.001, **** *p* < 0.0001; ns = not significant).

Having established that endogenous RTN3 is important for viral exosomal release, we next investigated whether augmenting or altering RTN3 can modulate this process. We overexpressed RTN3S and several deletion mutants in DENV-infected Huh7 cells to pinpoint domains required for viral cargo packaging into exosomes (Figure 3). The constructs included full-length RTN3S (RTN3S-FL) with a FLAG tag, two N-terminal truncation mutants (ΔN11 and ΔN45, lacking the first 11 or 45 amino acids, respectively), and a C-terminal truncation mutant (ΔC36, lacking the last 36 amino acids). All plasmid constructs were expressed to comparable levels, as confirmed by anti-FLAG immunoblots (Figure 3A). Full-length RTN3S-FL migrated at the expected size (~25 kDa), while the N- and C-terminal deletions showed slightly reduced molecular weight bands (consistent with their truncated sizes). DENV NS3 protein was present in all infected cultures, but its abundance differed depending on the RTN3 construct. Notably, cells overexpressing full-length RTN3S supported higher NS3 levels than empty-vector controls, whereas expression of the ΔC36 mutant correlated with a reduction in NS3 accumulation (Figure 3A).

We then quantified the impact of RTN3 constructs on exosome secretion. Overexpression of wild-type RTN3S dramatically boosted the release of exosomes from infected cells. Huh7 cells transfected with RTN3S-FL and infected with DENV secreted an approximately 5–7-fold greater number of exosome particles than infected cells carrying an empty vector (*p* < 0.0001) (Figure 3B). This hyper-secretion phenotype was partially reduced by small N-terminal deletions: the ΔN11 mutant still increased exosome output, although that the difference is not significant compared to empty vector, while the larger N-terminal deletion ΔN45) has elevated but not to the level of full-length RTN3S. Strikingly, the C-terminal truncation mutant ΔC36 failed to promote exosome biogenesis. In fact, exosome release in ΔC36-expressing cells was at or below the level of control infected cells (and significantly lower than that seen with full-length RTN3S, *p* < 0.001).

To determine how distinct RTN3S domains influence the incorporation of viral material into exosomes, we examined viral RNA transfer by RT-qPCR in recipient Huh7 cells after co-culture with purified exosomes (Figure 3C), followed by NS3 detection by Western blot (Figure 3D) following overexpression of full-length RTN3S (RTN3S-FL) and domain-deletion mutants (ΔN11, ΔN45, ΔC36) in DENV-infected Huh7 cells. RT-qPCR (Figure 3C) showed the highest DENV RNA in recipient cells exposed to exosomes from RTN3S-FL donors, minimal RNA with ΔC36, and intermediate levels with ΔN11 and ΔN45, indicating that the C-terminal tail is required for efficient viral transfer. Western blot analysis of recipient Huh7 cells after exosome co-culture revealed that exosomes from RTN3S-FL-expressing donors contained the highest levels of DENV NS3 protein, consistent with enhanced loading of viral cargo (Figure 3D). In contrast, exosomes from cells expressing the ΔC36 mutant showed negligible NS3 content, suggesting a severe defect in viral protein packaging. Exosomes from ΔN45- and ΔN11-expressing cells had moderately reduced NS3 levels compared to RTN3S-FL, with ΔN45 exosomes retaining slightly higher NS3 than ΔN11. Next, we assessed whether overexpression of RTN3 constructs affected exosome biogenesis and marker expression. Western blot analysis of donor cell lysates showed that TSG101 and CD63 levels were unchanged across all plasmid overexpression conditions (Figure S3A). Similarly, exosomes isolated from these cells displayed consistent expression of exosomal markers HSP70 and CD63, while the ER protein Calnexin was absent, confirming exosome purity (Figure S3B).

### 3.4. RTN3 Associates with DENV Replication Complexes and Viral RNA

**Figure 4:**
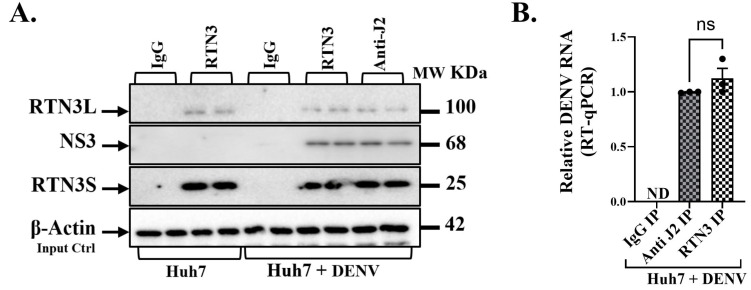
RTN3 is associated with Dengue viral double-stranded RNA and NS3 protein in infected Huh7 cells. (**A**) Western blot of the IP eluates from Huh7 + DENV lysates. Lanes: IgG IP (Huh7 + DENV), RTN3 IP (Huh7 + DENV), and anti-dsRNA (J2) IP (Huh7 + DENV), with input lysate as control. Blots were probed for RTN3L (~100 kDa), DENV NS3 (~68 kDa), and RTN3S (~25 kDa). (**B**) RT-qPCR detection of DENV genomic RNA in immunoprecipitated material from Huh7 cells 72 h after DENV infection. Cell lysates were immunoprecipitated with anti-RTN3 or anti-dsRNA (J2) antibodies; non-specific IgG was used as a negative control. Bars show relative enrichment of viral RNA in each IP (normalized to input). Data (**B**) are mean ± SEM of ≥3 experiments; significance by ANOVA or *t*-test (ns = not significant).

To elucidate the mechanism by which RTN3 facilitates viral packaging, we examined physical interactions between RTN3, viral proteins, and viral RNA in infected cells. Co-immunoprecipitation (co-IP) and RNA immunoprecipitation (RIP) experiments revealed that RTN3 is intimately associated with the DENV replication complex (Figure 4). Using an anti-RTN3 antibody, we immunoprecipitated RTN3 from lysates of DENV-infected Huh7 cells and probed for co-precipitating viral components. Western blot analysis showed that viral NS3 protein specifically co-immunoprecipitated with RTN3 in infected cells (Figure 4A). NS3 was readily detected in the RTN3 IP fraction from DENV-infected samples, whereas an isotype-matched IgG control IP pulled down no NS3. This indicates that RTN3 and NS3 form a complex or are part of the same membrane-bound assemblies during infection. In uninfected cells, RTN3 IP did not bring down NS3 (which is absent), confirming the specificity of the interaction under infection conditions (Figure 4A). Likewise, RTN3 itself was enriched in the RTN3-IP (and absent in IgG control), as expected. Interestingly, immunoprecipitation with a pan-dsRNA antibody (J2), which binds double-stranded RNA replicative intermediates, also pulled down RTN3 from infected cell lysates (Figure 4A).

Next, we directly probed whether RTN3 binds viral RNA by performing RIP followed by RT-qPCR for DENV RNA. Cell lysates were subjected to immunoprecipitation with anti-RTN3 or control IgG, and the pulled-down RNA was quantified. In DENV-infected samples, RTN3 immunoprecipitation brought down a significant amount of DENV genomic RNA, whereas the IgG control IP yielded none (Figure 4B). On average, RTN3-bound RNA contained DENV sequences at levels ~80–100% of those obtained by immunoprecipitating with the anti-dsRNA J2 antibody (the latter serves as a positive capture of replicating viral RNA). There was no detectable DENV RNA in RTN3 IP from uninfected cells.

### 3.5. Monocyte Infection Triggers RTN3-Linked Exosome Pathways and Immune Activation

**Figure 5:**
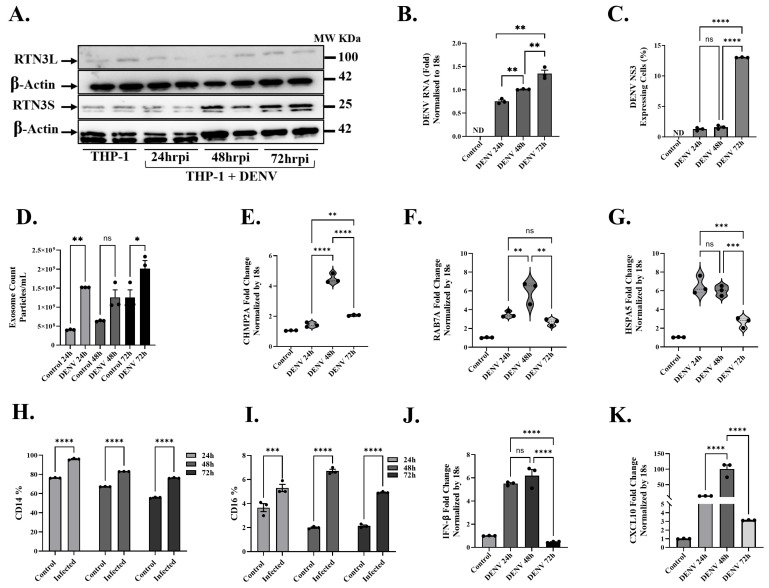
**Dengue virus induces RTN3 expression, exosome release, and innate immune responses in THP-1 monocytes.** (**A**) Western blot of THP-1 cells uninfected (Ctrl) or infected with DENV for 24, 48, or 72 h. Blots were probed for RTN3L (~100 kDa) and RTN3S (~25 kDa), with β-Actin (~42 kDa) as a loading control. (**B**) RT-qPCR of DENV genomic RNA in THP-1 cells (normalized to 18S) at each time point. (**C**) Flow cytometry of THP-1 cells for DENV NS3. (**D**) NanoFCM evaluation of exosomes in THP-1 culture supernatants with and without DENV infection. (**E**–**G**) RT-qPCR of THP-1 mRNA (normalized to 18S) for exosome biogenesis, trafficking genes, and chaperone protein: (**E**) CHMP2A, (**F**) RAB7A, and (**G**) HSPA5. (**H**,**I**) Flow cytometry quantification of monocyte markers. Bars show the percentage of cells expressing CD14 or CD16 in mock vs. DENV samples. (**J**,**K**) RT-qPCR of cytokine mRNAs: CXCL10 and IFN-β (normalized to 18S). Data (**A**–**I**) are mean ± SEM of triplicates; significance by ANOVA or *t*-test (ns = not significant, * *p* < 0.05, ** *p* < 0.01, *** *p* < 0.001, **** *p* < 0.0001).

To extend our findings to immune cells relevant to Dengue pathogenesis, we examined DENV infection in a human monocytic cell context. THP-1 monocytes were infected with DENV and monitored over 72 h (h) for RTN3 expression, exosome release, and innate immune responses (Figure 5). Kinetic analysis revealed a delayed but substantial activation of the RTN3-exosome pathway in these cells. Western blots showed that RTN3S protein was minimally expressed in resting THP-1 cells, but it became strongly induced by 72 h post-infection, with no noticeable changes in the RTN3L isoform (Figure 5A). By 48–72 h of infection, the RTN3S band intensity increased markedly compared to uninfected controls, suggesting a shift toward the short isoform as infection progressed. This timing corresponded with the accumulation of viral NS3-positive cells in the culture. Flow cytometry analysis indicated that only a small fraction (~1–2%) of THP-1 cells were NS3-positive at 24 h, increasing to ~4% at 48 h and ~13% by 72 h (Figure 5C). Thus, as the infection spread to more monocytes over time, RTN3S upregulation became pronounced in the cell population. RT-qPCR confirmed a gradual rise in DENV RNA levels in THP-1 cultures over 3 days (Figure 5B), though the increase was not exponential (consistent with limited infectivity of THP-1 and the low percentage of cells infected). By 72 h, viral RNA was less than 1-fold higher than at 24 h, paralleling the modest expansion of NS3+ cells.

Despite the relatively slow spread of infection, DENV elicited a significant enhancement of exosome secretion from monocytes at all time points. Even as early as 24 h, infected THP-1 cultures released more exosomes than mock-infected controls (Figure 5D). The exosome count in infected samples was ~1.5 × 10^9^ particles/mL at 24 h, compared to ~5 × 10^8^ in controls (≈33% increase, *p* < 0.001). At 48 h and 72 h, infected cells continued to secrete elevated exosome levels (~1.5 and 2 × 10^9^ particles/mL, respectively) relative to controls (~6 × 10^8^–~1.5 × 10^9^; *p* < 0.01 for 48 h, *p* < 0.05 for 72 h). DENV-infected monocytes also showed transcriptional upregulation of key regulators of the endosomal–exosomal pathway. Notably, mRNA levels of CHMP2A, a core component of the ESCRT-III complex essential for multivesicular body formation, rose significantly in infected THP-1 cells (Figure 5E). By 48 h post-infection, CHMP2A transcripts were ~5-fold higher than in uninfected cells (*p* < 0.0001). Similarly, at 72 h post-infection, the CHMP2A transcript levels were significantly higher than those of the controls (*p* < 0.001), but relatively low compared to the 48 h time point. Similarly, RAB7A, a small GTPase governing late endosome trafficking and maturation, was upregulated during infection (Figure 5F). RAB7A expression peaked at 48 h post-infection (~6-fold increase vs. control, *p* < 0.001) and remained ~2.5-fold elevated at 72 h (*p* < 0.01). The transient peak at 48 h might indicate an early cellular response to virus entry or an autophagic interaction, as Rab7A is known to modulate endolysosomal dynamics and exosome release. By 72 h, as infection became established, Rab7A levels tapered but stayed above control levels. We also observed a significant induction of HSPA5 (Grp78/BiP), an ER chaperone and unfolded protein response marker, in infected monocytes (Figure 5G). HSPA5 transcripts increased ~6-fold by 24 h (*p* < 0.0001) and remained elevated at 48 h without a significant change versus 24 h, before declining towards 72 h. Next, we assessed whether DENV-infected monocytes underwent phenotypic changes and mounted an innate immune response. We found that DENV induced a shift in monocyte subset markers over time. The proportion of CD14^+^ cells (monocyte marker) in culture decreased steadily with infection, dropping from ~95% at 24 h to ~ 75% by 72 h (*p* < 0.0001 at 72 h vs. control; Figure 5H). Conversely, the percentage of CD16^+^ monocytes (FCGR3A^+^, corresponding to intermediate monocytes) rose significantly in infected samples, especially at 48 h post infection (*p* < 0.0001 vs. control; Figure 5I).

By 48 h post-infection, THP-1 cells exhibited robust induction of cytokine and chemokine genes associated with antiviral and inflammatory responses. Interferon beta (IFN-β) itself was also induced, albeit with delayed kinetics (Figure 5J). Again, the levels of IFN-β transcripts were ~5-fold higher than baseline (*p* < 0.001) at 48 h, which correlates with the increased CXCL10 levels for this time point. CXCL10 (IP-10), a chemokine induced by interferon and a known marker of Dengue severity, was strikingly upregulated in infected monocytes (Figure 5K). CXCL10 mRNA increased ~6-fold at 24 h (*p* < 0.05) and continued to rise to ~100-fold above control by 48 h (*p* < 0.001).

### 3.6. Single-Cell Transcriptomics Links RTN3-High FCGR3A^+^ Monocytes with Exosome-Related Pathways and Clinical Severity in Dengue Virus Infection

**Figure 6:**
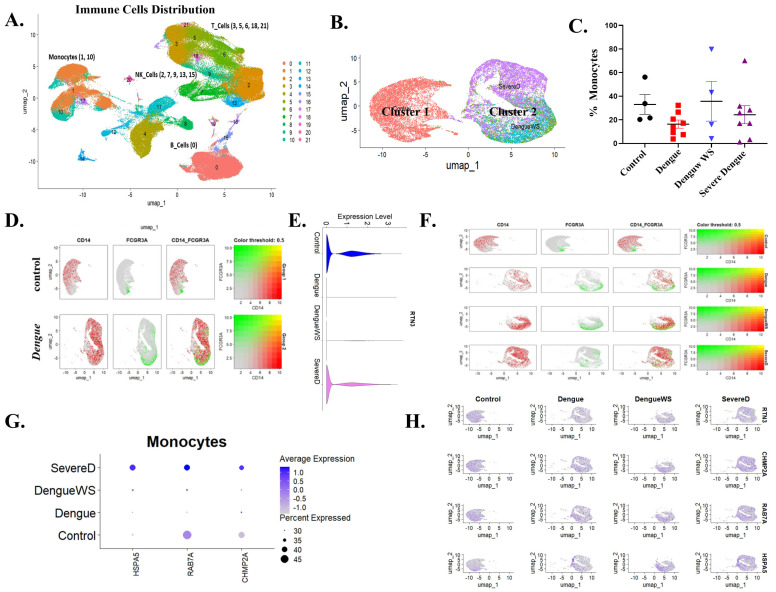
Single-cell transcriptomics of blood cells reveal monocyte subsets and RTN3-related signatures in Dengue patients. (**A**) UMAP projection of PBMCs from healthy controls and Dengue patients, colored by major immune cell types (monocytes, T cells, B cells, NK cells, etc.). Monocytes form a distinct cluster (red). (**B**) UMAP highlighting monocyte subclusters: cluster 1 (red) contains mainly healthy-donor cells, whereas cluster 2 (purple) is dominated by Dengue patient cells (including DFWS and severe cases). (**C**) Bar graph of the percentage of monocytes (CD14+ cells) among total PBMCs in each group (healthy control, Dengue fever (DF), Dengue with warning signs (DFWS), and severe Dengue (SD)). (**D**) Monocyte UMAP colored by expression of CD14 (red) and FCGR3A/CD16 (green) in healthy vs. Dengue samples. Classical monocytes (CD14^hi, red) and intermediate monocytes (FCGR3A^hi, green) are indicated. (**E**) Violin plots of RTN3 mRNA expression in monocytes from each group. Dengue patient monocytes, especially from severe cases, show higher RTN3 expression than controls. (**F**) Feature scatter plots on the monocyte UMAP showing FCGR3A (green) and CD14 (red) expression across disease conditions; yellow indicates co-expression. (**G**) Dot plot summarizing monocyte expression of HSPA5, RAB7A, and CHMP2A. Dot size corresponds to the proportion of cells expressing the gene; color indicates average expression. Dengue patient monocytes (Dengue, SevereD, and DengueWS) display larger and darker dots for these genes, indicating upregulation. (**H**) UMAP feature-plots of RTN3, CHMP2A, RAB7A, and HSPA5 in monocyte clusters across conditions, confirming stronger expression (blue) of all four genes in Dengue patient monocytes.

High-resolution, single-cell transcriptomics offers an unparalleled lens through which to dissect the cellular heterogeneity and pathway rewiring that underlie clinical deterioration in Dengue virus (DENV) infection. By resolving individual immune cells in patient blood, this approach permits direct linkage of gene-expression programs to disease severity. To evaluate the RTN3-centered, vesicle-trafficking mechanism during DENV infection, we leveraged a publicly available PBMC scRNA-seq dataset spanning healthy controls, uncomplicated Dengue, Dengue with warning signs (Dengue WS), and severe Dengue. We evaluated whether the RTN3-exosome axis is mobilized in vivo and whether its activation tracks with clinical outcome. Unsupervised UMAP projection of the complete immune compartment resolved major leukocyte lineages, with monocytes (clusters 1, 10) segregated from T, B and NK cells (Figure 6A). Re-clustering of the monocyte fraction uncovered two transcriptionally distinct groups: cluster 1 was dominated by control cells, whereas cluster 2 was enriched for Dengue samples, especially Dengue WS and severe Dengue (Figure 6B). Quantification revealed a reduction in the monocytes pool in Dengue patients compared to healthy individuals, with a slight increase observed in Dengue WS and a mild decrease in severe Dengue cases (Figure 6C). Marker co-embedding showed a shift from CD14^high^ classical monocytes towards FCGR3A [CD16^+^ non-classical and intermediate monocytes in Dengue (Figure 6D)], mirroring our THP-1 differentiation results. Violin plots confirmed a significant elevation of total RTN3 transcripts in Dengue monocytes versus controls (*t*-test, *p* < 0.001), with a severity-graded rise from mild to severe illness (Figure 6E). Single-cell transcriptomics (Figure 6F) demonstrated a progressive increase in intermediate monocytes (CD14++/FCGR3A^+^) across clinical severity groups, peaking in severe Dengue.

Consistent with an activated vesicle-trafficking, ESCRT-III component CHMP2A, late endosomal GTPase RAB7A, and ER-stress chaperone HSPA5, all required for infectious exosome production, showed progressive up-regulation across the clinical severity gradient, with peak induction in severe Dengue (Figure 6G). UMAP overlays corroborated the co-localization of these transcripts with RTN3-high monocyte islands (Figure 6H). Next, we analyzed transcriptional changes in HLA-A, HLA-B, ISG15, and CXCL10 across Dengue disease severity. Dot plot analysis revealed that the antigen presentation markers HLA-A and HLA-B were expressed at comparable levels in monocytes from healthy controls, Dengue infection, Dengue with warning signs, and severe Dengue patients (Figure S4A). In contrast, interferon-stimulated genes (ISG15 and CXCL10) showed marked upregulation in Dengue patient monocytes, with the strongest expression observed in Dengue with warning sign (Figure S4A). Additionally, violin plot analysis demonstrated that RTN3 expression was significantly elevated in intermediate monocytes from Dengue patients compared to healthy controls, with a trend toward higher expression in severe disease (Figure S4B).

## 4. Discussion

Exosomes are increasingly recognized as critical mediators of viral pathogenesis, functioning as non-canonical vehicles for the cell-to-cell spread of RNA viruses. In hepatitis C virus (HCV) infection, for example, serum-derived exosomes were shown to carry replication-competent HCV RNA and assemble a complex of viral RNA with host factors (Ago2, miR-122, HSP90) that could infect naïve hepatocytes in a receptor-independent manner [29]. These findings underscore that exosomes can subvert classical entry pathways to disseminate infection. By analogy, recent works in flaviviruses (including Dengue virus [DENV]) and coronaviruses (SARS-CoV-2) have revealed that infected cells release EVs containing viral RNAs and proteins, which can promote transmission and modulate immunity. For instance, exosomes from DENV-infected cells have been found to contain full-length DENV genomes and viral proteins, enabling “stealth” infection of new target cells while evading neutralizing antibodies [21]. However, until now, the cellular mechanisms that drive the packaging of viral components into infectious exosomes have remained poorly defined. Here, we identify Reticulon-3 (RTN3), an ER-membrane curvature protein, as a key regulator of infectious exosome biogenesis in DENV infection, thereby filling a major gap in our understanding of how infectious vesicles are generated during RNA virus infection.

**Figure 7:**
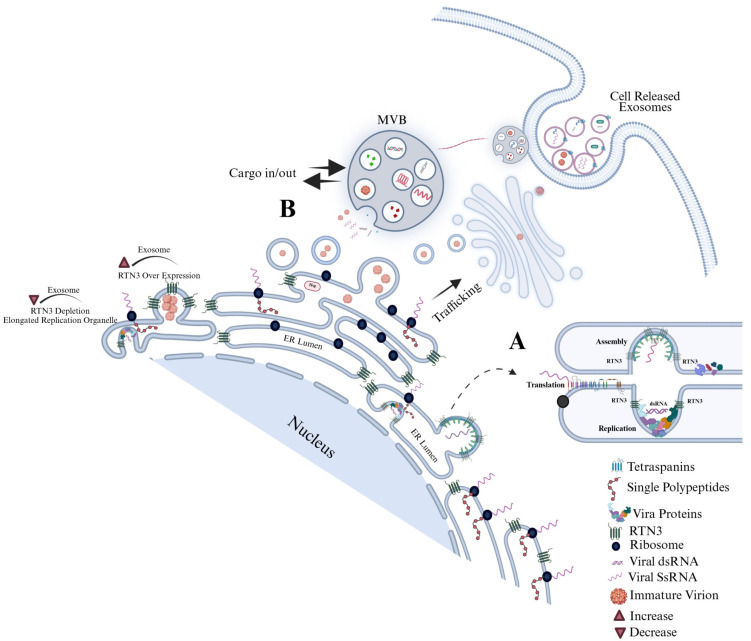
Dengue virus manipulates the endoplasmic reticulum (ER) landscape through the short Reticulon-3 isoform (RTN3S) to generate infectious exosomes. Following DENV entry, positive-strand genomic RNA is translated on rough ER membranes. RTN3S (dark green hairpin elements) accumulates at the peri-nuclear ER and sculpts it into bulb-like invaginations toward the ER lumen. These curved subdomains recruit ribosomes (navy spheres), newly synthesized viral polyprotein (dashed red coils), and non-structural replication factors (-colored icons), forming sealed single-membrane vesicle packets that entrap double-stranded replicative DENV RNA (magenta helices). (**A**) Magnified inset in the graphic highlights how RTN3S helices directly clasp dsDENV RNA, creating a selective ribonucleoprotein scaffold that favors viral, over cellular, cargo. (**B**) Viral particles bud from the ER and converge with early endosomes to seed multivesicular bodies (MVBs). Inside the maturing MVB, they become intraluminal vesicles destined to leave the cell as exosomes. A bold “cargo in/out” arrow at the MVB boundary mirrors RTN3S’s dual role: it both guides viral material into the endosomal lumen and licenses its export once the MVB fuses with the plasma membrane. The released exosomes are depicted dispersing from the cell, each carrying replication-competent DENV RNA, non-structural proteins, and occasional immature virions, an immune-evasive inoculum primed for cell-to-cell spread. Modulating RTN3S levels alters every step in this pathway. RTN3S over-expression (upper left) intensifies ER curvature, increases the number of replication organelles, and drives a surge in cargo-rich exosome release. Conversely, RTN3S depletion (lower left) yields elongated, poorly scissioned replication tubules, drastically reduces vesicle trafficking to MVBs, and suppresses exosome output. These opposing phenotypes, annotated directly on the schematic, underscore RTN3S as a gatekeeper that links flavivirus RNA replication to selective exosome biogenesis and extracellular dissemination.

Our study shows that DENV infection robustly upregulates RTN3 (particularly the short isoform, RTN3S, while the long isoform RTN3L remains unchanged) in host cells. It is plausible that the virus preferentially induces RTN3S because the smaller isoform’s streamlined structure is more easily accommodated at sites of membrane curvature, making it especially effective at packaging viral components into budding exosomes. We also observed that RTN3 localizes to sites of viral replication and budding. We found that RTN3 co-immunoprecipitated with DENV non-structural protein 3 (NS3) and double-stranded viral RNA, suggesting a direct interaction with the viral replication complex (analogous to RTN3’s known binding of HCV RNA). Functionally, CRISPR-mediated RTN3 knockdown profoundly reduced the release of exosomes and nearly abolished the export of DENV NS3 and viral RNA into extracellular vesicles. Conversely, overexpressing RTN3S strongly increased exosome secretion and the infectivity of DENV-containing exosomes. Using a series of RTN3S deletion mutants, we mapped the effect on the carboxy-terminal amphipathic helices of the protein: deletion of the C-terminal 36 amino acids abrogated the ability of RTN3 to enhance exosome release or to load infectious cargo, whereas N-terminal deletions had lesser effects. These gain and loss-of-function studies definitively establish RTN3 as an ER-shaping factor whose membrane-bending activity is co-opted by DENV to bud vesicles containing viral genomes. Importantly, these findings mirror and extend prior work in HCV [28] showed that RTN3 KD decreased the number of infectious HCV-bearing exosomes, while RTN3 overexpression increased them [28]. Thus, RTN3 emerges as a novel selective cargo-sorting scaffold that directs viral RNA and proteins into secreted exosomes. In mechanistic terms, RTN3’s C-terminal helices likely induce membrane curvature and recruit the nascent viral RNP complex into intraluminal vesicles of multivesicular bodies, coupling ER morphological remodeling to EV biogenesis (Figure 7). This mode of action is distinct from, yet complementary to, classical vesicle-sorting pathways [31,32,33].

RTN3’s role can be compared with other known ER- or EV-related host factors. The double-stranded RNA-binding protein Staufen1 (STAU1) is a cytosolic regulator that has been shown to bind viral RNAs and promote HCV and influenza replication [34]. Like RTN3, STAU1 can interact with viral genomic RNA and replication complexes, but STAU1 functions primarily in RNA transport and translational regulation, not membrane shaping. In DENV, STAU1 might theoretically help escort viral RNA, but it would not directly induce vesicle formation as RTN3 does. The ESCRT-III component CHMP2A has been reported to bind Dengue virus RNA and participate in the budding of virus particles and vesicles. [35] However, CHMP2A’s role in EV formation is broad and not specific to RNA cargo selection [36,37]. In contrast, RTN3 appears to be an RNA-sensing factor that specifically enriches viral replication products into vesicles. Similarly, small GTPases like RAB7A regulate endosomal fate: normally, RAB7A drives late endosomes toward lysosomal degradation and thereby limits exosome secretion [38,39,40]. By contrast, RTN3 overexpression effectively increases vesicle output even in the face of high RAB7A, indicating that RTN3 can override endolysosomal routing to favor secretion.

Another ER chaperone, HSPA5 (BiP/GRP78), is induced by DENV infection and is critical for the proper folding of viral proteins [41]. HSPA5 was identified in recent high-throughput screens as a proviral factor in flaviviruses. We observed that DENV infection of monocytes upregulated HSPA5, along with other ER-stress markers, and these factors were prominent in exosome-associated protein networks. However, unlike HSPA5’s role as a general chaperone and stress sensor, RTN3 provides a structural scaffold. TMEM41B (an ER transmembrane protein important for lipid mobilization and autophagy) is another proviral host factor for DENV, as part of the replication membrane platform. Whereas TMEM41B facilitates membrane lipid flux, RTN3 specifically sculpts the curved ER membranes into vesicular buds [42]. DNAJC3 (p58IPK), an ER luminal co-chaperone involved in the unfolded protein response, is also implicated in viral infections, but its function is again chaperone-like rather than membrane-bending [43]. In summary, RTN3 is unique among these factors: it is an ER-membrane remodeler that directly connects viral replication sites to the extracellular vesicle biogenesis pathway.

Our results also highlight how RTN3-driven EV production tangibly alters immune responses. In DENV-infected monocyte cultures, we observed not only a surge in exosome release but also a phenotypic shift in monocyte subsets. DENV infection induced expansion of CD16^+^ intermediate monocytes (CD14^+^CD16^+^), both in vitro and as reflected in human Dengue patient single-cell data. This population was identified previously in Dengue patients [44] and is associated with stimulating plasmablast and antibody responses. Our flow cytometry data showed an increase in CD16 expression on THP-1 cells, mostly at 48 h of infection. Our scRNA-seq analysis revealed that patient monocytes in the Dengue cohort co-express FCGR3A (CD16) with RTN3. Transcriptionally, infected monocytes upregulated several exosome/ER-related genes: CHMP2A and RAB7A were induced, and HSPA5 remained elevated. These changes suggest a coordinated antiviral stress program: Elevated CHMP2A and RAB7A may reflect enhanced endomembrane turnover, while HSPA5 indicates ER stress from viral protein load. Concomitantly, infected monocytes exhibited elevated CXCL10 and IFN-β, consistent with an interferon-driven antiviral state. Notably, exposing naïve monocytes to exosomes from DENV-infected Huh7 cells recapitulated this activation profile, inducing CD16 upregulation and a surge in IFN-β and CXCL10, underscoring that infectious exosomal cargo alone can trigger these innate immune responses in bystander cells. Thus, DENV not only hijacks RTN3 to make infectious exosomes, but the resulting vesicle traffic and ER remodeling feed back into immune activation pathways. It is plausible that infectious exosomes (laden with viral RNA/NS proteins) contribute to innate sensing in bystander cells, or conversely, could temper immune detection by cloaking viral RNA within host membranes.

These findings have significant implications for DENV pathogenesis and potential therapies. By packaging viral genomes into exosomes, DENV may amplify infection foci and subvert neutralizing antibodies, much as was seen with HCV. Targeting RTN3 or its interactors could therefore block a parallel transmission route. For example, we note that HSP90 inhibitors impaired exosome-mediated HCV spread [28]. Given that RTN3 complexes with HSP90-bound RNA [28], similar strategies might limit DENV EV infectivity. More broadly, viruses that exploit exosomes (including flaviviruses like Zika and even SARS-CoV-2) may share components of this pathway. Indeed, emerging evidence indicates that cells infected by SARS-CoV-2 release exosomes containing viral RNA and proteins [45], suggesting commonality in exosomal export of viral cargo across disparate RNA viruses. Indeed, SARS-CoV-2-infected cells release exosomes containing viral RNA and proteins, potentially promoting spread while evading immune recognition. Our discovery of RTN3 as an EV regulator suggests new therapeutic angles: modulation of ER curvature proteins or inhibition of specific cargo-loading domains could attenuate exosome-mediated infection. Notably, RTN3 also influences autophagy and ER-phagy [46], hinting that autophagy-modulating drugs (e.g., V-ATPase inhibitors) might indirectly affect EV release as we observed in HCV models.

In conclusion, this work advances the field by revealing a direct mechanistic link between the ER membrane-shaping machinery and the generation of infectious viral exosomes. By identifying RTN3 and its functional domains as critical for packaging DENV RNA into EVs, we address a long-standing knowledge gap in extracellular vesicle biology during viral infection. Our integrated analysis, from molecular virology to single-cell immunophenotyping, highlights how RTN3-driven vesicles reshape the host response and viral spread. These insights open new research directions, such as exploring RTN3 inhibitors or studying ER curvature proteins in other virus systems, intending to disrupt EV-mediated pathogenesis and enhance antiviral strategies.

## Figures and Tables

**Table 1:** Sequences of Oligonucleotides.

Primer	Sequence 5′ to 3′
RTN3S	F: GGAGAGATGTGAAGAAGACTGCC R: AGATCCTGAAGCTGATGGTGA
RTN3L	F: GTAGGGAGGCTAAAACTGCA R: CTCCTGAAACTTTGGATGGAGA
DENV	F: TTATCAGTTCAAAATCCAATGTTGGT R: AGGAGGAAGCTGGGTTGACA
CHMP2A	F: GAAGACGCCAGAGGAGCTACTGC R: GCTTGGCCATCTTCTTAATGTCTGC
RAB7A	F: GTCGGGAAGACATCACTCA R: CTAGCCTGTCATCCACCAT
HSPA5	F: GGGAGGTGTCATGACCAAAC R: GCAGGAGGAATTCCAGTCAG
CXCL10	F: GTGGATGTTCTGACCCTGCT R: GGAGGATGGCAGTGGAAGTC
IFNB	F: TGGGAGGCTTGAATACTGCCTCAA R: TCCTTGGCCTTCAGGTAATGCAGA
18S	F: GTAACCCGTTGAACCCCATT R: CCATCCAATCGGTAGTAGCG
gRNA cloning verification	F: AGCTGCAGCTCTTCGTC R: TCGGCGGCCACTCAGTC
gRNA sequence targeting RTN3S	CTCGGCTCCGAAGGACGACG

## Data Availability

Data is contained within the article or Supplementary Material: The original contributions presented in this study are included in the article/Supplementary Material. Further inquiries can be directed to the corresponding author.

## Supplementary Materials

The following supporting information can be downloaded at: https://www.mdpi.com/article/10.3390/v17091238/s1, **Figure S1. Differential expression of RTN3 isoforms in Huh7 cells following DENV infection.** Western blot densitometry quantification of RTN3 isoforms in control and DENV-infected Huh7 cells. (**A**) RTN3S protein levels were significantly upregulated in Huh7-DENV cells compared to uninfected controls. (**B**) RTN3L protein levels showed no significant change between groups. Band intensities were normalized to β-Actin. **Figure S2. Nano-flow cytometry analysis of extracellular vesicles (EVs) derived from DENV-infected Huh7 cell culture supernatants (CCS).** (A) Analysis of unstained CCS: the top panel shows the side scatter (SS-H) distribution, while the bottom panel displays a dot plot comparing SS-H and fluorescence in the FITC-A channel, serving as a baseline reference. (B) CCS samples stained with AF647-conjugated anti-CD63: the top panel presents the SS-H histogram, and the bottom panel shows a dot plot of SS-H versus AF647 fluorescence (PC5-A channel), with defined gates identifying CD63-positive (P1) and CD63-negative (P2) populations. (C) CD63-immunopurified exosomes from CCS, treated with RNase, and stained with AF647 anti-CD63. The top panel illustrates the SS-H histogram, and the bottom panel shows a dot plot of SS-H versus AF647 fluorescence, indicating a higher concentration of CD63⁺ EVs following purification and enzymatic treatment. All analyses were performed on the NanoFCM N30E using consistent acquisition settings, with particle size estimated within the 40–200 nm range based on calibration with silica beads. Each condition was tested in triplicate. **Figure S3. Overexpression of RTN3 constructs does not alter exosomal marker expression.** (**A**) Western blot analysis of Huh7 cell lysates transfected with Empty Vector, RTN3S-FL, ΔN11, ΔN45, or ΔC36 probed for TSG101 and CD63. Comparable expression levels were observed across all conditions. (**B**) Exosomes isolated from the same donor cells were analyzed for exosomal markers HSP70 and CD63. The ER protein Calnexin was used as a negative control and was absent from exosome preparations, confirming exosome purity. **Figure S4. Monocyte expression of antigen presentation genes, interferon-stimulated genes, and RTN3 across dengue severity.** (**A**) Single-cell transcriptomics dot plot showing monocyte expression of HLA-A, HLA-B (MHC class I), ISG15, and CXCL10 in healthy controls, Dengue infection (Dengue), Dengue with warning signs (DengueWS), and severe Dengue (SevereD). Dot size reflects the proportion of monocytes expressing each gene, while color intensity indicates average expression levels. HLA-A and HLA-B were similarly expressed across all groups, whereas ISG15 and CXCL10 were strongly upregulated in Dengue patients, particularly in severe Dengue. (**B**) Violin plot showing RTN3 expression in intermediate monocytes across patient groups. RTN3 levels were elevated in Dengue patients compared to controls, with higher expression observed in severe Dengue.
