# Supplementary material for: Integrative Mechanistic Studies Identify Reticulon-3 as a Critical Modulator of Infectious Exosome-Driven Dengue Pathogenesis
Source: Viruses. 2025 Sep 13;17(9):1238. doi: 10.3390/v17091238 (PMC12474469; doi:10.3390/v17091238)
Supplement: Supplementary file 1 [file viruses-17-01238-s001.zip › viruses-3868360-supplementary.pdf]

viruses

Article

Integrative Mechanistic Studies Identify Reticulon-3 as a Critical Modulator of Infectious Exosome-Driven Dengue Pathogenesis

Razieh Bitazar [1,2], Clinton Njinju Asaba [1], Saina Shegefti [1], Tatiana Noumi [1], Julien Van Grevenynghe [1], Salim T. Islam [1,2], Patrick Labonté [1] and Terence Ndonyi Bukong [1*]

[1] Armand-Frappier Santé Biotechnologie Research Center, Institut National de la Recherche Scientifique, Laval, QC, Canada
[2] PROTEO, the Quebec Network for Research on Protein Function, Structure, and Engineering, Montréal, QC, Canada
* Correspondence: author: Terence Ndonyi Bukong, E-mail: terencendonyi.bukong@inrs.ca

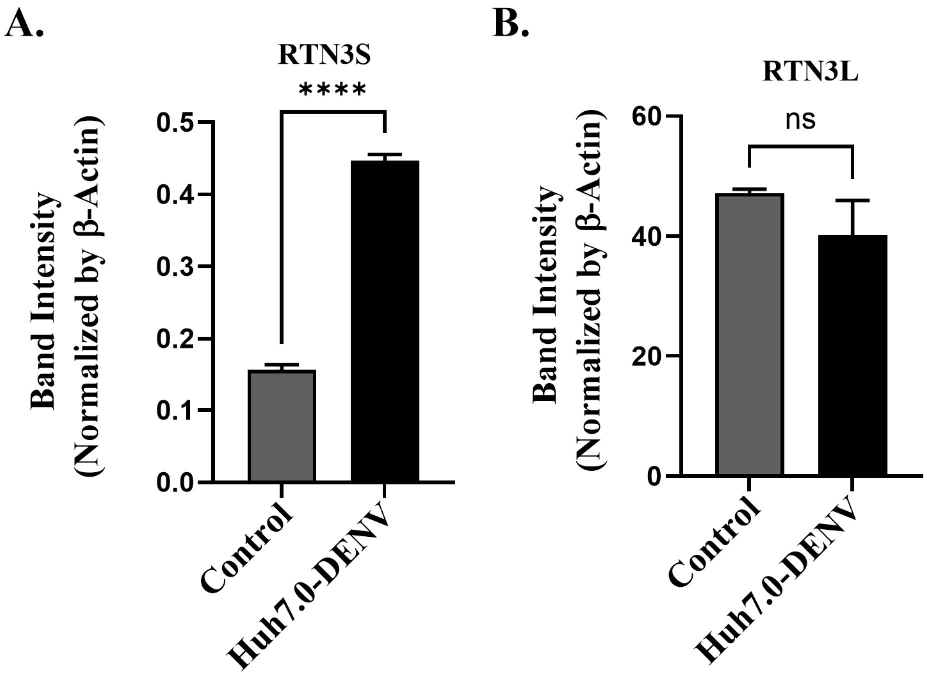

Figure S1. Differential expression of RTN3 isoforms in Huh7 cells following DENV infection. Western blot densitometry quantification of RTN3 isoforms in control and DENV-infected Huh7 cells. (**A**) RTN3S protein levels were significantly upregulated in Huh7-DENV cells compared to uninfected controls. (**B**) RTN3L protein levels showed no significant change between groups. Band intensities were normalized to β-Actin.

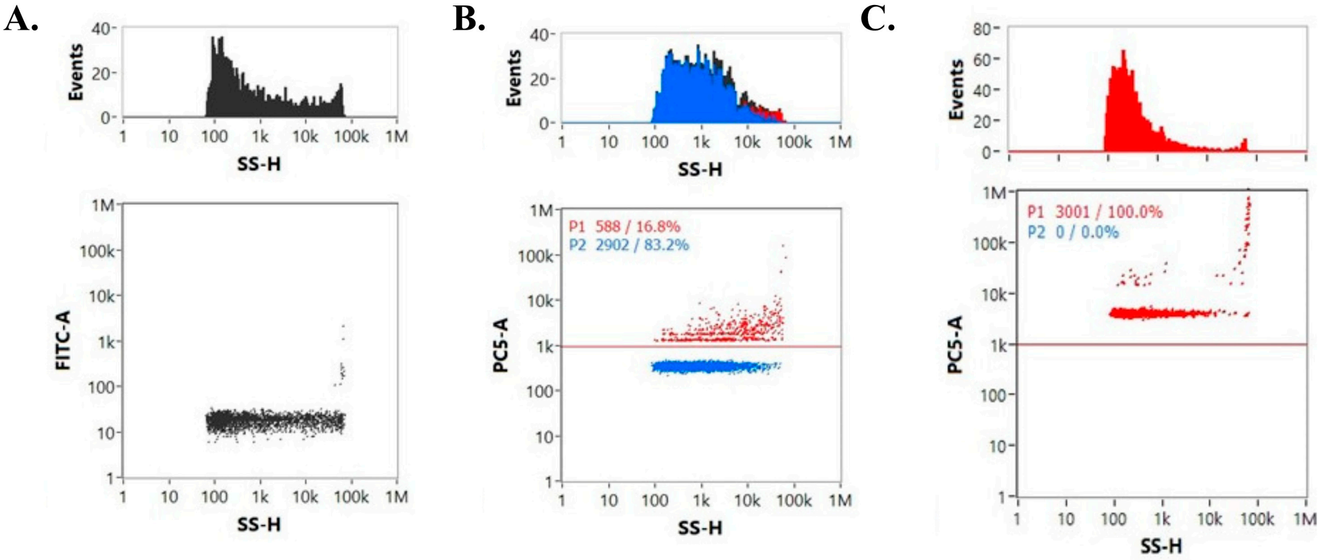

Cell Culture Supernatant (CCS)
from DENV-infected -Huh7 cells (Unstained)

Cell Culture Supernatant (CCS)
from DENV-infected-Huh7 cells
probed with Anti-CD63 Alexa Flour 647

Immuno-purified and RNase Treated Exosomes
from Cell Culture Supernatant (CCS)
of DENV-infected-Huh7 cells
probed with Anti-CD63 Alexa Flour 647

Figure S2. Nano-flow cytometry analysis of extracellular vesicles (EVs) derived from DENV-infected Huh7 cell culture supernatants (CCS). (A) Analysis of unstained CCS: the top panel shows the side scatter (SS-H) distribution, while the bottom panel displays a dot plot comparing SS-H and fluorescence in the FITC-A channel, serving as a baseline reference. (B) CCS samples stained with AF647-conjugated anti-CD63: the top panel presents the SS-H histogram, and the bottom panel shows a dot plot of SS-H versus AF647 fluorescence (PC5-A channel), with defined gates identifying CD63-positive (P1) and CD63-negative (P2) populations. (C) CD63-immunopurified exosomes from CCS, treated with RNase, and stained with AF647 anti-CD63. The top panel illustrates the SS-H histogram, and the bottom panel shows a dot plot of SS-H versus AF647 fluorescence, indicating a higher concentration of $CD63^{+}$ EVs following purification and enzymatic treatment. All analyses were performed on the NanoFCM N30E using consistent acquisition settings, with particle size estimated within the 40-200 nm range based on calibration with silica beads. Each condition was tested in triplicate.

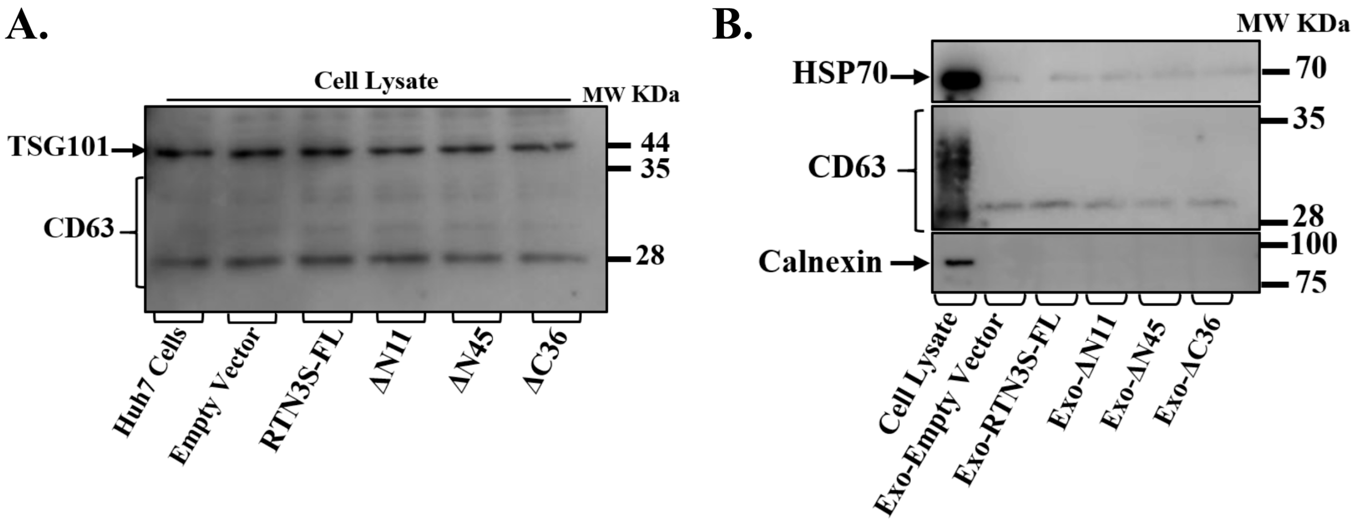

Figure S3. Overexpression of RTN3 constructs does not alter exosomal marker expression. (**A**) Western blot analysis of Huh7 cell lysates transfected with Empty Vector, RTN3S-FL, ΔN11, ΔN45, or ΔC36 probed for TSG101 and CD63. Comparable expression levels were observed across all conditions. (**B**) Exosomes isolated from the same donor cells were analyzed for exosomal markers HSP70 and CD63. The ER protein Calnexin was used as a negative control and was absent from exosome preparations, confirming exosome purity.

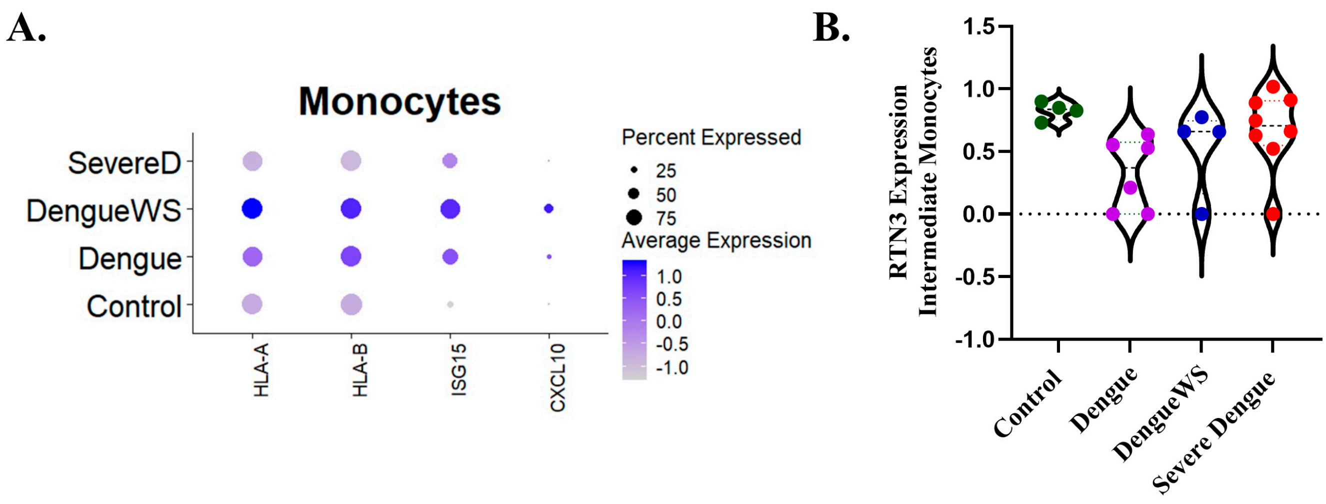

Figure S4. Monocyte expression of antigen presentation genes, interferon-stimulated genes, and RTN3 across dengue severity. (**A**) Single-cell transcriptomics dot plot showing monocyte expression of HLA-A, HLA-B (MHC class I), ISG15, and CXCL10 in healthy controls, Dengue infection (Dengue), Dengue with warning signs (DengueWS), and severe Dengue (SevereD). Dot size reflects the proportion of monocytes expressing each gene, while color intensity indicates average expression levels. HLA-A and HLA-B were similarly expressed across all groups, whereas ISG15 and CXCL10 were strongly upregulated in Dengue patients, particularly in severe Dengue. (**B**) Violin plot showing RTN3 expression in intermediate monocytes across patient groups. RTN3 levels were elevated in Dengue patients compared to controls, with higher expression observed in severe Dengue.
